# Tonsillar hyperplasia and recurrent tonsillitis: clinical-histological correlation

**DOI:** 10.5935/1808-8694.20130108

**Published:** 2015-10-08

**Authors:** Luciana Guedes Vilela Reis, Élia Cláudia de Souza Almeida, Juliano Carvalho da Silva, Gilberto de Araújo Pereira, Valdirene de Fátima Barbosa, Renata Margarida Etchebehere

**Affiliations:** a MSc in Pathology - Federal University of Triangulo Mineiro (UFTM), Uberaba - MG; MD. Otorhinolaryngologist.; b PhD in Pathology, Post-doctorate in Sciences - Federal University of Triangulo Mineiro (UFTM), Uberaba - MG; Graduate Student.; c Biologist; Lab Technician - Surgical Pathology Service - University Hospital - Federal University of Triangulo Mineiro (UFTM), Uberaba - MG.; d PhD in Statistics; Professor, Department of Biostatistics - Federal University of Triangulo Mineiro (UFTM), Uberaba - MG.; e MSc in Computer Science and Computational Mathematics - Statistics.; f PhD in Pathology and Forensic Pathology; Pathologist - Surgical Pathology Service - University Hospital - Federal University of Triangulo Mineiro (UFTM), Uberaba - MG. Federal University of Triangulo Mineiro.

**Keywords:** palatine tonsil, surgical pathology, tonsillectomy

## Abstract

Hypertrophy and recurrent tonsillitis are common indications of tonsillectomy. However, the pathological reports are similar, regardless of clinical aspects.

**Objective:**

Search for histopathological changes that differentiate palatine tonsils operated because of hypertrophy vis-à-vis those operated because of recurrent tonsillitis.

**Method:**

A prospective cross-sectional descriptive study involving 46 children divided into group I - 22 with hypertrophy; and group II - 24 with recurrent tonsillitis, in the period between 2010 and 2012, in a public hospital. We evaluated clinical and histopathological features (lymph follicles, germinal centers, fibrosis, necrosis, reticulation, infiltration by plasma cells and neutrophils).

**Results:**

The patients' ages ranged between 2 and 11 years (5.17 ± 2.28). In group I, half of the patients had had the latest infection at seven months or more, and all with obstruction degree greater than 3 (≥ 50%). In group II, all had had the latest infection at less than seven months, and most with obstruction degree below 4 (≤ 75%). There was a statistically significant difference in the degree of obstruction (*p* = 0.0021) and number of germinal centers (*p* = 0.002) was higher in group I.

**Conclusion:**

This study suggests that the number of germinal centers is the only histopathological criterion that can be used to differentiate the two groups.

## INTRODUCTION

Palatine tonsils are part of the Waldeyer's lymphatic ring, responsible for the first line of defense against pathogens because it is located at the entrance of the air and digestive tracts^1^. The lymphatic ring is also composed of the pharyngeal, lingual and torus tubarius tonsils, and the lymphatic tissue scattered throughout the posterior oropharyngeal wall, with the function of collecting antigenic information^2^.

The lymphatic tissue is not usually apparent in early childhood, but it gradually evolves with hypertrophy and hyperplasia and reaches its largest size between 2 and 5 years of age. Its involution, which has unknown cause, starts at puberty. In adulthood, there is only a small amount of lymphatic tissue remaining^3^.

The full role the ring plays in human physiology and immunology and its effects on the immune system both local and systemic are not yet completely understood^4^.

Tonsillectomy is one of the most performed surgeries in ENT practice, particularly in children, due to the intense activity and large amounts of lymphatic tissue existing in this period of their lives.

Despite the progress made in the various fields of medicine, infections of the upper respiratory tract such as acute otitis media, acute sinusitis, sore throat and tonsillar hypertrophy are highly prevalent diseases and their treatment is responsible for a large portion of healthcare costs^5^.

In many children, adenotonsillar hypertrophy is associated with sleep breathing disorders, ranging from obstruction - leading to snoring, all the way to obstructive sleep hypopnea apnea syndrome (OSAHS)^6^, ^7^, ^8^.

In clinical practice, we have noticed that histopathological diagnosis in most tonsillectomies is “lymphoid tissue hyperplastic-reactive state” or “nonspecific lymphatic hyperplasia”, regardless of the clinical and surgical indication of the patient be associated with hypertrophy or recurrent tonsillitis.

Thus, we consider it important to assess the simple histopathological findings to morphologically differentiate hypertrophy with consequent obstruction from recurrent tonsillitis and correlate them with clinical signs.

With this study, we aimed at comparing tonsil histopathology in children with recurrent tonsillitis and tonsillar hyperplasia submitted to tonsillectomy.

## METHOD

This study was approved by the Ethics Committee of the Institution under protocol number 1674 on August 27, 2010. The Informed Consent Form (ICF) was signed by the legal guardian of the child. The study sample consisted of all children undergoing tonsillectomy in our institution, and the guardian consented on participating in the study and signed the consent form. Therefore, there was no statistical calculation of sample size and probability criterion for selecting the children.

A form on the history of each patient was filled, containing data such as age, gender, race, indication for surgery, frequency of tonsillitis occurrence, the date of the last tonsillitis episode and the degree of oropharyngeal obstruction by the tonsils - classified into grades 0 to IV, according to the scheme proposed by Brodsky (1989)^9^.

This is a descriptive prospective cross-sectional study. We chose to evaluate only the palatine tonsils of children with an indication for tonsillectomy for recurrent tonsillitis or hypertrophy, according to well defined clinical criteria. Children have a high activity and amount of lymphatic tissue, and both hypertrophy and recurrent tonsillitis are very frequent^6^, ^7^, ^9^.

The selected patients were children with previous surgical indication made by one of four assisting otolaryngologists to be submitted to tonsillectomy according to well-defined clinical criteria: relevant respiratory obstruction (intense nighttime snoring, sleep apnea and compensatory mouth breathing) and no improvement with medical therapy or recurrent tonsillitis according to the Paradise criteria^10^.

The exclusion criterion was the surgical indication for both conditions simultaneously. Although some patients with recurrent tonsillitis also had hypertrophic palatine tonsils had no obstruction to justify surgery, and patients with hypertrophy could have tonsillitis, however, not of the repetition type.

The sample consisted of 46 children submitted to tonsillectomy in the period of 2010 a 2012. The histological criteria analyzed were number of follicles and type of lymphatic follicles, number of germinal centers, fibrosis, necrosis permeating neutrophils in crypt epithelium and plasmocyte infiltration around the crypts.

Patients were divided into groups according to the indication for surgery: group I with 22 children, and group II with 24 children with surgical indications for tonsillar hypertrophy, with consequent respiratory obstruction or recurrent tonsillitis, respectively.

The tonsils removed were identified as left and right and fixed in 10% formaldehyde. After surgical specimen fixation we did a macroscopic description (weight, measurements, features) and material cleavage. We made 3 mm parallel cross-sections in the specimens and at least two sections were included for each tonsil. The material was subsequently processed, embedded in paraffin - we made histological cross-sections of about 5 micrometers in thickness, stained according to the hematoxylin-eosin (HE) technique for general specimen evaluation; Masson trichrome and reticle to assess fibrosis and the support reticular framework, respectively.

The slides were analyzed by a pathologist and an otolaryngologist simultaneously. We evaluated the presence of the criteria used by Lopes et al.^11^: lymph follicles, germinal centers, fibrosis and necrosis.

The lymph follicles were analyzed in HE staining, in ordinary light microscopy at 40x magnification. First, they were classified into primary and secondary groups and distributed in the following groups: 0 - predominance of primary follicles; 1 - predominance of secondary follicles and 2 - similar proportions between primary and secondary follicles.

The follicles were also counted in five fields, also at 40x magnification, which subjectively had a higher number of follicles. We counted the mean number of follicles and the cases were divided into two groups: 1 - less than 25 follicles per field of 40x and 2 - 25 or more follicles per 40x field. The count in five fields was defined after observing that, in most cases, from this number of fields there was no large variation in the number of follicles. Moreover, in some cases it was not possible to count a larger number of fields.

The germinal centers were analyzed in HE staining, in ordinary light microscopy at 100x. After an overall assessment we counted five fields of 100x, which subjectively had a higher number of germinal centers. We calculated the mean value and the cases were distributed in subgroups: 1 - less than six germinal centers per field and 2 - more than six germinal centers per field.

We also looked for necrosis and fibrosis in 100x magnification, besides neutrophil permeation in crypt epithelium in 400x magnification. After identifying the most intense focus, fibrosis, necrosis and neutrophil permeation were subjectively classified into: 0 - absent; 1 - mild; 2 - moderate and 3 - intense.

We also analyzed the reticulation and infiltration by plasmocytes around the crypts with a 400x magnification, similar to what was done by Endo & Vassalo^12^. After identifying the most intense focus, they were also subjectively classified into: 0 - absent; 1 - mild; 2 - moderate and 3 - intense.

In the statistical analysis to detect differences in clinical and epidemiological characteristics in the numerical form [Age (years), rate of infection episodes per year; degree of obstruction by tonsils (%), time elapsed since the last infection (month) and tonsil weight (g)], between groups: Hypertrophy (I) and Recurrent tonsillitis (II); we used the Mann-Whitney test due to data non-normality verified by the Shapiro-Wilks test.

When considering the variables in its categorical form, due to the low frequencies in crossings between the variables of interest, we considered the regrouping of some categories. This procedure decreases the level of accuracy of the information; however, it is an alternative to the robustness of the statistical test utilized. Even so, we continue with low frequencies; thus the associations of interest were analyzed by Fisher's exact test. For instance, in the case of three or more layers, the sum of two of them, for example, for the level of microscopic change, were added to the level 1 and level 2 (0 - absent, 1 - mild, 2 - moderate and 3 - intense), to the level of obstruction caused by the tonsils we added levels 2 and 3 (1 - 0 to 25%; 2 - 26 to 50%; 3 - 51 to 75%; and 4 - 76 a 100%); and for the number of infection episodes we combined levels 3 and 5 (3 - three episodes per year for three consecutive years; 5 - five episodes per year for two consecutive years and 7 - seven episodes per year). All tests were performed considering a significance level of 5%.

## RESULTS

We evaluated and compared the epidemiological, clinical and histopathological characteristics of both groups. In the Hypertrophy group (I), most subjects (81.8%) were white, with a slight predominance of females (54.5%). In group II, most were white (91.7%) and there was a slight predominance of males (58.3%). The groups were homogeneous for age (*p* = 0.8), which ranged between 2 and 11 years (5.17 ± 2.28) (Table 1).

**Table 1:** Distribution of patients according to gender, color, number of tonsillitis episodes per year, oropharynx obstruction and time elapsed since the last infection and the Hypertrophy (I) and Recurrent Tonsillitis (II) Groups - 2011-2012.

		Group				Fisher-Exact *p*-value
		I		II		
		N	%	N	%	
Gender	Males	10	45.5	14	58.3	0.560
	Females	12	54.5	10	41.7	
	Total	22	100	24	100	

Skin color	White	18	81.8	22	91.7	0.400
	Not white	4	18,2	2	8.3	
	Total	22	100	24	100	

Number of tonsillitis episodes	3	-	-	5	20.8	-
	5	-	-	9	37.5	
	7	-	-	10	41.7	
	Total	-	-	24	100	

Degree of obstruction (%)	25-50	0	0.0	10	41.7	0.07#
	5-75	15	68.2	12	50	
	76-100	7	31.8	2	8.3	
	Total	22	100	24	100	

Time of the last infection (month)	1 a 6	10	50.0	24	100	< 0.0001*
	7 a 12	10	50.0	0	0,0	
	Total	20	100	24	100	

Hypertrophy;

Recurrent tonsillitis;

* Significant association;

# Added to the categories of the degree of obstruction (25%-50% and 51%-75%) for performing the Fisher's exact test; as for the number of episodes, there are three: three episodes per year for three consecutive years; 5: five episodes per year for two consecutive years and 7: seven episodes per year.

Clinically, in all children in the recurrent tonsillitis group (II), the last infection occurred less than seven months in the past, and 41.7% had seven or more infections per year, the vast majority had obstruction degree ≤ 75%. In group I, half of the children had had the last infection last 7 months or more in the past and all had more than 50% obstruction of the oropharynx by the palatine tonsils. We found a statistically significant higher degree of oropharyngeal obstruction by the palatine tonsils (70.5% ± 11.9%, *p* = 0.0021) and longer duration (in months) since the last infection (6.4% ± 4.1%, *p* = 0.0002) in the hypertrophy (I) group, as expected (Table 1).

Regarding the histopathological characteristics, the groups were homogeneous for tonsil weight (*p* = 0.2942). Only the number of germinal centers was statistically significant (*p* = 0.002) between the two groups, being higher in the Hypertrophy Group(I).

The type and number of lymph follicles, fibrosis, necrosis, permeation by neutrophils in the epithelium of the crypts, reticulation and infiltration by plasmocytes around crypts showed no statistically significant differences between the two groups (Table 2).

**Table 2:** Distribution of patients by the categorical variables: lymph follicles and germinal centers; fibrosis; necrosis; neutrophil permeation around the tonsil crypt epithelium and collagen fibrosis in the Hypertrophy Group (I) and Repetition Tonsillitis (II), and the level of these changes - from 2011 to 2012.

Level of Microscopic Alteration												
	Group	0		1		2		3		Total		Fisher's Exact *p*-value
		N	%	N	%	N	%	N	%	N	%	
FOL-LINF	I	-	-	2	9.1	20	90.9	-	-	22	100	0.140
	II	-	-	7	29.2	17	70.8	-	-	24	100	

CEN-GERM	I	-	-	1	4.6	21	95.4	-	-	22	100	0.002*
	II	-	-	11	45.8	13	54.2	-	-	24	100	

Fibrosis	I	-	-	20	90.9	2	9.1	-	-	22	100	0.220
	II	-	-	24	100	0	0.0	-	-	24	100	

Necrosis	I	21	95.5	1	4.5	-	-	-	-	22	100	1.000
	II	23	95.8	1	4.2	-	-	-	-	24	100	

NEU-CRIPT	I	6	27.3	9	40.9	7	31.8	-	-	22	100	0.750
	II	8	34.8	11	47.8	4	17.4	-	-	23	100	

RET-CRIPT	I	-	-	3	13.6	16	72.7	3	13.6	22	100	1.000
	II	-	-	9	37.5	13	54.2	2	8.3	24	100	

INF-PLASM	I	-	-	11	50.0	11	50.0	-	-	22	100	1.000
	II	-	-	12	50.0	11	45.8	1	4.2	24	100	

FB-COLA	I	-	-	18	81.8	4	18.2	-	-	22	100	0.410
	II	-	-	22	91.7	2	8.3	-	-	24	100	

I: Hypertrophy, II recurrent tonsillitis;

FOL-LINF.: Number of lymph follicles; CEN-GERM: Number of germinal centers; NEU-CRIPT: Permeation by neutrophils in the epithelium around the crypts; RET-CRIPT: reticulation around the crypts; INF-PLASM: plasmacytic infiltration around crypts, FB-COLA: collagen fibrosis, in the case of three or more levels of histopathological changes, we summed up levels 1 and 2 (0: absent, 1: mild, 2: moderate, 3: severe).

* Significant association;

Two patients (4.3%) from the total, both belonging to group I (9.09%) exhibited non-invasive colonies of bacteria, morphologically consistent with Actinomyces Sp.

## DISCUSSION

According to Alcantara et al.^7^, tonsillectomy is the most commonly performed surgical procedure in pediatric patients. The ages of the patients in this study coincide with the age group where hypertrophy and tonsillitis are more intense and frequent^3^, ^13^.

The highest degree of obstruction by palatine tonsil hypertrophy in the Hypertrophy Group was statistically significant, as expected. Hypertrophy, with consequent respiratory obstruction, is among the absolute indications for tonsilectomy^6^.

There was a higher concentration of germinal centers in the Hypertrophy Group. The presence of the germinal center indicates that the lymphoid follicle is very active in producing lymphocytes^14^. The greater immune activity and the presence of intensively active germinal centers, with or without acute or chronic infection are features found in children, in accordance with the findings of Lopes et al.^11^.

We also found - though not statistically significant, a higher concentration of lymph follicles in the Hypertrophy Group. We know that tonsil size and volume vary according to age and the presence of prior infection and/or inflammation^15^. It is likely that since there was no difference in tonsil weight between the two groups, the lymph follicles and germinal centers of the Hypertrophy Group were smaller or there was less connective or lymphatic tissue in the extrafollicular area located between them. Our study showed no difference in the connective tissue and did not evaluate the lymphatic tissue in the extrafollicular area.

There may be differences between the antigens involved in the two entities, and in the Hypertrophy Group there is a higher stimulus for greater B cell differentiation. Another possibility would be to have a difference in immune response in both groups compared to the same antigen. In addition, the two groups could represent different poles of the same disease, which still display the intermediate cases in which the two manifestations associated with Hypertrophy and Recurrent Tonsillitis.

Colonies of bacteria morphologically consistent with Actinomyces sp. were found only in two of the patients in the Hypertrophy Group, representing 4.34% of these cases. There is still controversy in the literature regarding the association between Actinomyces sp. and palatine tonsil hypertrophy. A low incidence of this association was described by Dell'Aringa^16^, suggesting that Actinomyces sp. may not be related to the palatine tonsil hypertrophy. Different results were published by Bhargava et al.^17^ and Kearns et at.^18^ - they found a high incidence of Actinomyces sp. in children undergoing tonsillectomy for obstructive reasons compared with other ones operated because of recurrent infections. In our study, although only two cases had bacterial colonies morphologically consistent with Actinomyces SP, both belonged to the Hypertrophy Group and accounted for 9.09% of these patients.

Some authors suggest that persistent antigenic stimulation by these and other pathogens could explain the development of hypertrophy that causes symptomatic airway obstruction^18^.

It would be interesting to invest in simple histopathology findings which enable the classification and differentiation of hypertrophy and recurrent tonsillitis - providing better clinical correlation and better diagnosis.

## CONCLUSION

The type and number of lymph follicles, fibrosis, necrosis, neutrophil permeation in crypt epithelium, reticulation and infiltration by plasmocytes around the crypts, although presenting differences between hypertrophy and recurrent tonsillitis, these were not statistically significant.

The number of germinal centers was found to be the sole criterion able to differentiate the palatine tonsils of children operated by hypertrophy from recurrent tonsillitis. When there are more than six germinal centers per field at 100x magnification, it is a case of tonsillar hypertrophy.
